# Occupational kneeling and squatting: development and validation of an assessment method combining measurements and diaries

**DOI:** 10.1007/s00420-014-0946-5

**Published:** 2014-05-24

**Authors:** Dirk M. Ditchen, Rolf P. Ellegast, Tom Gawliczek, Bernd Hartmann, Monika A. Rieger

**Affiliations:** 1 Institute for Occupational Safety and Health of the German Social Accident Insurance, Alte Heerstr. 111, 53757 Sankt Augustin, Germany; 2 ARBMEDERGO, Hamburg, Germany; 3 Institute of Occupational and Social Medicine and Health Services Research, University Hospital of Tuebingen, Tuebingen, Germany; 4 Department of Occupational Medicine and Environmental Health, Institute of General Practice and Family Medicine, University of Witten/Herdecke, Witten, Germany

**Keywords:** Posture capturing, Diary, Exposure, Kneeling and squatting, Knee osteoarthritis

## Abstract

**Objectives:**

As knee-straining postures such as kneeling and squatting are known to be risk factors for knee disorders, there is a need for effective exposure assessment at the workplace. Therefore, the aim of this study was to develop a method to capture knee-straining postures for entire work shifts by combining measurement techniques with the information obtained from diaries, and thus avoiding measuring entire work shifts. This approach was applied to various occupational tasks to obtain an overview of typical exposure values in current specific occupations.

**Methods:**

The analyses were carried out in the field using an ambulatory measuring system (CUELA) to assess posture combined with one-day self-reported occupational diaries describing the durations of various work tasks. In total, 242 work shifts were measured, representing 81 typical tasks from 16 professions. Knee-straining postures were analysed as daily time intervals for five different postures. The accuracy of the method was examined by comparing the results to measurements of entire work shifts.

**Results:**

Unsupported kneeling was the most widely used knee posture in our sample (median 11.4 % per work shift), followed by supported kneeling (3.0 %), sitting on heels (1.1 %), squatting (0.7 %), and crawling (0.0 %). The daily time spent in knee-straining postures varied considerably, both between the individual occupations, within an occupation (e.g. parquet layers: 0.0–88.9 %), and to some extent even within a single task (e.g. preparation work of floor layers (22.0 ± 23.0 %). The applied measuring method for obtaining daily exposure to the knee has been proven valid and efficient randomly compared with whole-shift measurements (*p* = 0.27).

**Conclusions:**

The daily degree of postural exposure to the knee showed a huge variation within the analysed job categories and seemed to be dependent on the particular tasks performed. The results of this study may help to develop an exposure matrix with respect to occupational knee-straining postures. The tested combination of task-based measurement and diary information may be a promising option for providing a cost-effective assessment tool.

## Introduction

Knee-straining postures such as kneeling, squatting, sitting on heels, and crawling are known to be risk factors for injuries and diseases such as osteoarthritis of the knee or meniscal tears. Numerous studies provide evidence supporting this relationship, especially in an occupational context (Cooper et al. 1994; Coggon et al. 2000; Sandmark et al. 2000; Seidler et al. 2008; Klussmann et al. 2010). Apart from the individual health impairment, the associated economic impact of absenteeism and the cost of treatment due to knee disorders are considerable. For example, the German Statutory Health Insurance companies reported an absenteeism rate in the year 2003 of 2.71 million days due to knee osteoarthritis and 4.40 million days due to unspecific knee damage (Liebers and Caffier 2009). To address the problem of occupational kneeling and squatting in terms of prevention, in epidemiological studies, and during occupational diseases procedures, the detailed knowledge of daily exposure is crucial. To quantify this exposure, different methods are available from very basic questionnaires to sophisticated technical solutions. In studies examining dose–response relationships between knee-straining work activities and degenerative knee disorders, retrospective exposure assessment has usually been based on self-reports (Felson et al. 1991; Vingard et al. 1991; Coggon et al. 2000; Sandmark et al. 2000; Seidler et al. 2008; Muraki et al. 2009; Klussmann et al. 2010). However, as various studies have shown, the validity of self-reports, specifically in this field, might be questionable (Baty et al. 1986; Burdorf and Laan 1991; Viikari-Juntura et al. 1996; Ditchen et al. 2013).

Alternatively, prospective methods of exposure assessment such as workplace observations, video-recordings, or exposure measurements that provide more accurate data are applied in assessing knee-straining postures. Yet, they are only rarely used, potentially as a result of the associated technical and financial efforts and the question of optimal cost efficiency by weighing up precision and costs against each other (e.g. Trask et al. 2014). Consequentially, in studies using these methods, exposure assessment is often conducted for only short sequences and focuses on small participant groups. For example, Kivimäki et al. (1992) investigated knee disorders of floor layers, carpet layers, and painters (*N* = 35) by videotaping working tasks including kneeling and squatting with a total observation time of 12 h. A similar approach was used in a Danish study (Jensen et al. 2000a) on kneeling and squatting of carpenters and floor layers. The authors filmed short working sequences and extrapolated the duration of knee-straining postures to an entire work shift. This procedure may have led to overestimation of the daily knee-loading, as critically stated by the authors in a recent publication (Jensen et al. 2010).

To avoid this source of bias, Burdorf et al. (2007) examined the entire work shift to investigate the effects of mechanised equipment on physical load among road workers and floor layers (*N* = 59) in the Netherlands. A complex method of exposure assessment was applied, with work postures (e.g. kneeling and squatting) being measured by an ambulant-monitoring equipment system using accelerometry combined with a hand-held computer for real-time observations by the researchers. On the one hand, such technical solutions deliver valid exposure data of whole work shifts. On the other hand, this approach must be seen as an exception as it requires enormous effort in terms of time, technical and human resources.

Beyond different tools for exposure assessment as described above, there may be different approaches to estimate the exposure in a study population either on an “individual” level, i.e. for each subject separately, or using a “group approach” where all subjects of an exposure group are assigned the group mean (Svendsen et al. 2005). Additionally, there is the question of whether exposure assessment should be designed on a “task-based” or in a more “naive” or “job-based” manner (Mathiassen et al. 2003, 2005). Both aspects will not be addressed in this article, but all these different approaches require valid exposure data as a basis for their different strategies.

The aim of this study was to develop an employable method to capture knee-straining postures for entire work shifts in the field by combining measurement techniques with the information delivered by diaries. As knee-straining postures were to be recognised automatically in the measurement data, the accuracy of this automated posture recognition by the evaluation software was examined first (*pretest*). Second, within in a *validation study,* the results of the combined assessment were compared with whole-shift measurements. Third, the feasibility of the combined approach for field studies was shown. In this *main study*, exposure data for various occupational tasks were collected to show the nature of occupational knee-loading and to provide an overview of typical postural exposure levels to the knee in current occupations in Germany.

## Methods

### Knee-straining postures

**Fig. 1:**
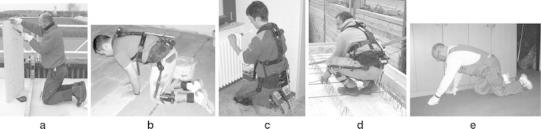
Knee-straining postures: **a** unsupported kneeling (roofer); **b** supported kneeling (tiler), **c** sitting on heels (installer), **d** squatting (reinforcement ironworker); and **e** crawling (floor layer). Subjects **b**–**d** are equipped with the CUELA measuring system

We focussed on five postures that are described as risk factors for the development of knee osteoarthritis, according to the definition of the respective occupational disease listed in the German schedule of occupational diseases (No. 2112) (BMGS 2005). These included unsupported kneeling (one or both knees on the ground without supporting the trunk with the upper extremities), supported kneeling (one or both knees on the ground with additional support of the upper extremities), sitting on heels (both knees on the ground and contact between heels and backside), squatting (no knee on the ground), and crawling (moving on all four extremities) (Fig. 1). For identification of the particular postures, knee flexion was defined as the angle between the imaginary axis of the thigh and the front side of the lower leg; standing with straight legs was defined as neutral position. Kneeling or squatting with thigh-calf-contact (Caruntu et al. 2003) was defined as deepest flexion with a knee angle of 155° (maximum flexion, Zelle et al. 2009).

### Posture capturing

**Fig. 2:**
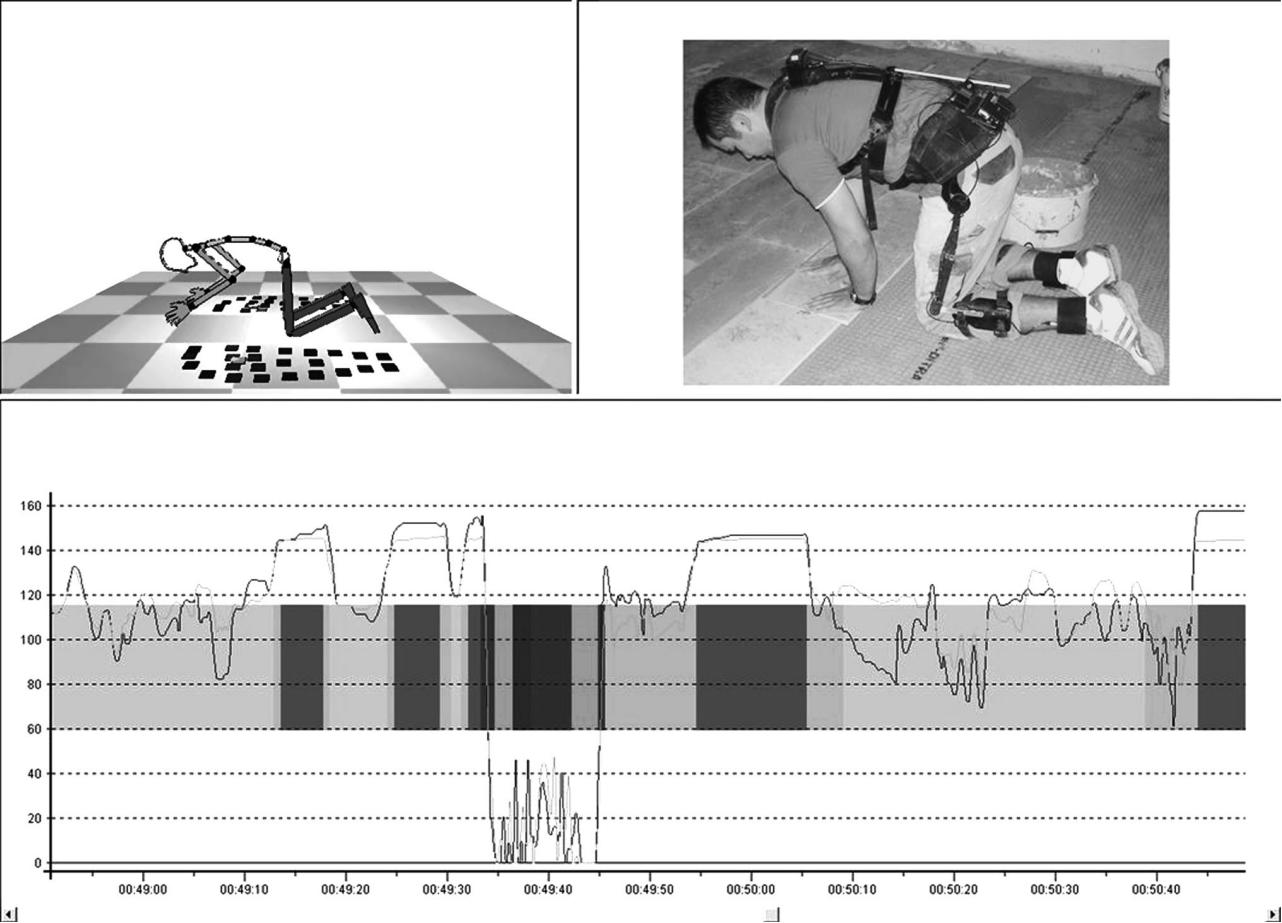
Screenshot of the analysis software depicting a measuring-based vector puppet (*top left*), the synchronised video sequence (*top right*), angular-time-graphs of the measured knee flexion (for both knees), and automatic identification codes for various postures (*colour bars*, *bottom*)

Posture capturing was performed using the ambulant measuring system *CUELA* (German abbreviation for “computer-assisted recording and long-term analysis of musculoskeletal loads”). The system has been used for several years in various studies to assess physical stress in numerous occupations and settings (e.g. Ellegast et al. 2009; Freitag et al. 2007, 2012; Glitsch et al. 2007). The system consists of gyroscopes, inclinometers, and potentiometers that are integrated in a belt system to be fixed on a person’s clothing (Fig. 1, b, c, and d). This system allows for time-continuous recording of body angles and the calculation of postures and movements of the trunk (thoracic spine, lumbar spine) and lower limb (hip and knee joints) with a sample rate of 50 Hz. A rechargeable battery pack runs the system allowing the subject to do his work independently and in a usual manner. All sensor data are directly logged on the system itself and saved on a memory card for subsequent IT-analyses. Every measurement is accompanied by video-recording, allowing a parallel view on the measured exposure and the real working situation after synchronisation of sensor and video data within the appropriate analysis software (Fig. 2, top left and right). The video data are only used for verification purposes and do not contribute to the posture analysis.

The software features an automated recognition for various body postures and movements and allows for the analysis of occurrence, frequency, duration and dynamics of the defined postures (unsupported kneeling, supported kneeling, sitting on heels, squatting, and crawling), and measured variables (e.g. knee flexion, Fig. 2, bottom).

All measurements were performed by experienced technical services of the Statutory Accident Insurance companies, applying a total of ten measuring systems used in parallel at various locations in Germany.

### Task modules or typical shifts

For all examined occupations, a board of technical experts of the German Statutory Accident Insurance defined typical tasks in which knee-straining postures were assumed to occur frequently and which were usually carried out for a whole work shift, for example tilers’ work can be separated into floor tiling, wall tiling, et cetera. These single tasks and their concomitant activities such as preparation and clearance work, breaks, and driving time were combined as *task modules* or *typical shifts*. It was planned to measure at least three work shifts performed by different workers per task module to capture inter-individual variations. In reality, working conditions limited this protocol to a total of 81 task modules, and 30 modules (=37.0 %) were measured less than three times (15 modules (=18.5 %) were measured just once; another 15 modules (=18.5 %) were measured just twice).

### Sampling strategy

As one of the aims of the study was to assess daily exposure of a task module without measuring the entire work shift, it was necessary to obtain the full information about all single tasks occurring during a shift and to prioritise tasks to be measured based on the criteria of them containing knee-straining postures. For this purpose, in preparation for the measuring day, information regarding the tasks was collected from the participating enterprises and a measuring plan was developed. Finally, this plan was completed by the subjects themselves reporting all tasks, concomitant activities, and breaks of the day using a sort of diary. For example, when investigating floor layers’ task module *laying carpet*, we were measuring the single tasks *application of glue* and *laying carpet* in the morning, and he reported all tasks and breaks happening in the afternoon (Table 1). By combining the information from the diary with the actually measured data that could be copied to cover all respective task periods, a reconstruction of the work shift was developed (Table 1, last column).

**Table 1:** Example of a diary and measuring schedule of a floor layer with two measuring samples used for reconstruction of a whole shift (task module: laying carpet; M1 and M2 = measurement samples)

Time	Task (derived from the diary)	Measurement	Kneeling/squatting	Reconstruction
07.00–07.30	Approach (driving)		–	Non relevant
07.30–08.00	Preparation of worksite		–	Non relevant
08.00–08.30	Application of glue	M1	×	M1
08.30–10.30	Laying carpet	M2	×	M2
10.30–11.00	Application of glue		×	M1 copy
11.00–12.30	Laying carpet		×	M2 copy
12.30–13.00	Break		–	Break
13.00–13.30	Preparation work		–	Non relevant
13.30–14.00	Application of glue		×	M1 copy
14.00–15.30	Laying carpet		×	M2 copy
15.30–16.00	Clearing of worksite		–	Non relevant

Non relevant = none of the defined knee-straining postures occurred

As a result, the reconstructed work shift could consist of four different time periods: single tasks accompanied by original measurements, single tasks with time-related copies of measurement data, non relevant parts (i.e. concomitant activities), and breaks. The median duration of the original measurements per work shift was 2.2 h (0.5–7.7 h), and 530 h in total were used for analysis.

### Pretest

The accuracy of the CUELA system and the sensors used in the system has been validated in earlier studies with a multiple-camera motion analysis system (Ellegast 1998; Schiefer et al. 2011). In addition, the automatic identification of the five knee-straining postures by the analysis software (Fig. 2) was validated by comparing the duration of the single knee-straining activities as derived from the automatic analysis of the measurement data with the video-taped time intervals of knee-straining postures in the first measuring sample of every single occupation (*n* = 16) by one observer (DMD).

### Validation study

To validate the specific method of shift reconstruction performed in this study, a validation study was initiated comparing the “reconstructed” exposure with the results of “total shift measurements”. The test consisted of 14 work shifts (eight service technicians, four ramp agents, and two nursery nurses). In each case, posture capturing with CUELA for an entire work shift of seven to 8 h in total was performed.

As a result, we could indicate the time proportions per day spent in the five different knee-straining postures (“measured shift”). Additionally, for every single work shift, a schedule was filled out containing the time periods of all single tasks that have been performed during the shift (similar to Table 1). From these schedules, two or three typical task periods of about 30–50 % of the whole working time were selected and defined as being representative for the whole work shift.

After the measurement, the measuring data of these time periods (“snippets”) were extracted by one of the authors (TG) from the whole measuring data and used as sample files to reconstruct a new working shift by copying and transferring them according to the schedule filled out before (“reconstructed shift”). Thus, we were able to compare the knee-straining postures of the “measured shift” with the “reconstructed shift” by descriptive and nonparametric statistics.

### Study sample

The validation study was conducted with 14 subjects with a mean age of 35.0 years (SD = 12.5) in three different occupations (eight male service technicians, four male ramp agents, and two female nursery nurses).

The main study involved a total of 16 different occupations known as professions at risk of developing knee osteoarthritis or other knee pathologies (Coggon et al. 2000; Vingard et al. 1991; Kivimäki et al. 1992; Jensen et al. 2000a; Wickström et al. 1983). From the respective industry sectors, 110 employers were contacted by the German Statutory Accident Insurance and all agreed to participate in the study with 213 male employees from these enterprises volunteering to participate in the measurements. Their mean age was 35.5 years (SD = 11.3), and all subjects were skilled craftsmen. As 17 subjects participated in more than one measurement, a total of 242 work shifts were analysed (Table 2).

**Table 2:** Occupations with number of subjects (and their average age), work shifts, and task modules in the study

Occupation	*N*	Age (years)	Work shifts (*n*)	Task modules (*n*)
Floor layers	15	43.9 (10.8)	16	4
Installers/plumbers	34	36.6 (13.7)	40	12
Mould makers	4	29.5 (10.3)	4	1
Painters and decorators	18	32.7 (13.2)	19	7
Parquet layers	14	32.1 (9.5)	28	7
Pavers	7	35.6 (4.8)	7	3
Pipe layers	9	37.3 (12.8)	9	4
Ramp agents	8	28.5 (6.6)	8	2
Reinforcing ironworkers	6	33.2 (5.8)	6	2
Roofers	34	34.8 (10.9)	36	14
Screed layers	17	35.7 (10.2)	20	7
Shipyard workers	6	32.5 (7.7)	6	3
Stone layers	15	39.0 (8.7)	15	5
Tilers	19	35.2 (12.2)	20	8
Truck tarp makers	4	37.5 (11.3)	5	1
Welders	3	32.0 (19.1)	3	1
Total	213	35.5 (11.3)	242	81

Values for age are mean values (SD)

### Statistical analysis

The validity of the automatic posture identification in the pretest was confirmed using linear regression and *t* test for paired samples. For the comparison of the measured and reconstructed work shifts in the validation study, the Wilcoxon signed-rank test (paired samples) and Spearman’s rank correlation coefficient were used. The time spent in knee-straining postures in different task modules is depicted by descriptive statistics (arithmetic means, standard deviations, and box-plots showing percentiles 5, 25, 50, 75, and 95).

## Results

### Pretest

The dependent t test for paired samples showed no significant differences (*p* = 0.1705) between measured and manually reconstructed exposure to the knee time intervals. Further analyses showed a strong coefficient of determination for both measurements and video-recordings (*R* ^2^ = 0.8913). Only for the steep-roofing work task, a high percentage of “knee-supporting working position” (Jensen et al. 2000b) was automatically categorised as “standing” and therefore had to be modified manually for analysis. After exclusion of this task, the coefficient of determination between the two methods improved further (*R* ^2^ = 0.9978).

### Validation study

**Fig. 3:**
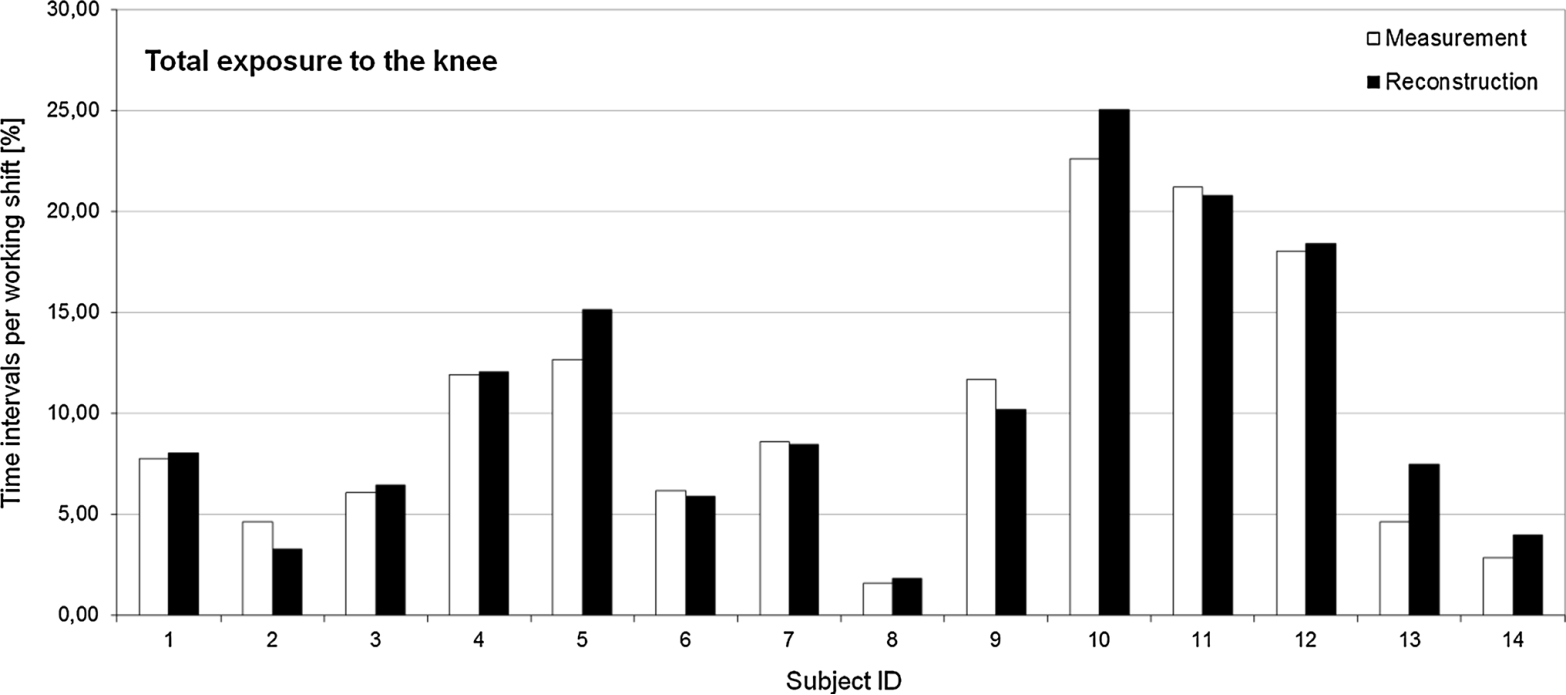
Pilot study: comparison of measured (*white*) and “reconstructed” (*black*) exposure to the knee: time intervals spent in knee-straining postures during an entire work shift (*n* = 14) in three occupations (subject *ID 1–8* service technicians, *ID 9–12* ramp agents, *ID 13–14* nursery nurses)

Figure 3 depicts the time spent in knee-straining postures (unsupported kneeling, supported kneeling, sitting on heels, squatting, and crawling) during an entire work shift, both originally measured and reconstructed, for each of the 14 subjects from the three different occupations. The average time spent in knee-straining postures was 10.02 ± 6.68 % per work shift for the measurements and 10.50 ± 6.97 % for the reconstructions. The absolute deviations between measured and reconstructed daily knee strain (time percentages) ranged from 0.06 to 2.86 % with an average deviation of 0.48 %. An equal distribution of small over- and underestimations was found (57–43 %, respectively). Thus, the results of both methods seem to be very similar, and there is no visible trend for a false estimation of the degree of exposure by the reconstruction method.

This apparent similarity is supported by the results of the Wilcoxon signed-rank test, which shows no significant differences between the two methods for any of the knee-straining postures; *p* values ranged from 0.21 (sitting on heels) to 1.00 (crawling), with *p* = 0.27 for knee-straining postures in total.

For Spearman’s rank correlation coefficient, very good correlations were found between both methods for all analysed forms of exposure. The calculated values were between 0.90 (squatting) and 0.98 (supported kneeling), with 0.97 for knee-straining postures in total and *p* < 0.0001 for all values.

### Main study: postural exposure to the knee

**Fig. 4:**
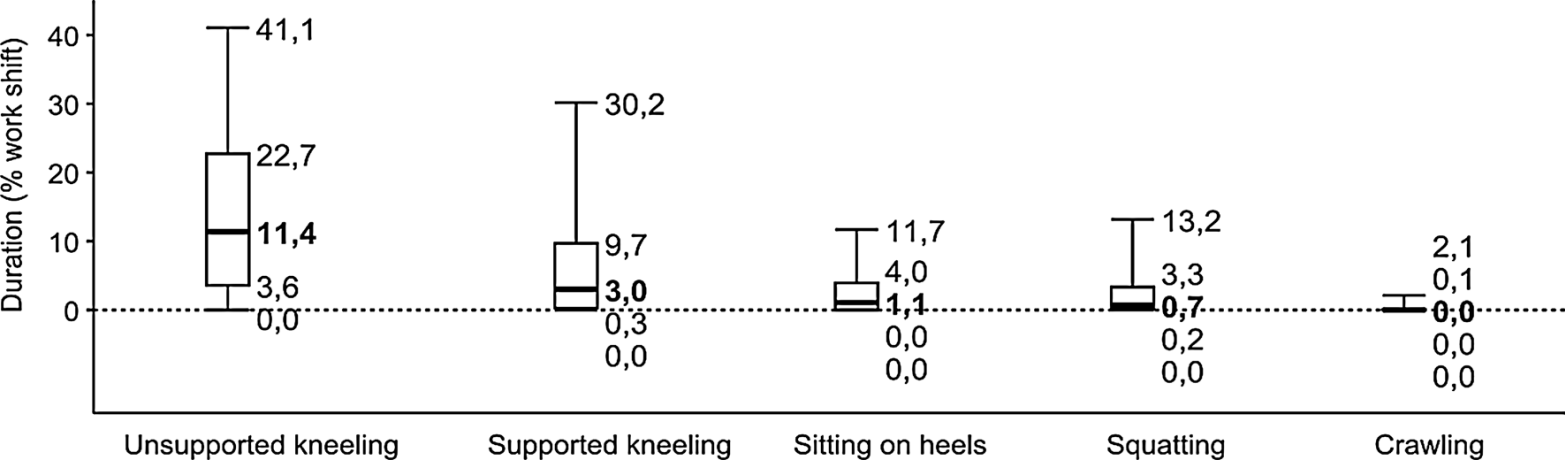
Distribution of daily time intervals spent in five different knee-straining postures over all measurements (*box-plots* showing percentiles 5, 25, 50, 75, and 95; *N* = 242 work shifts)

Figure 4 shows the distributions of daily time intervals of the analysed postures over all examined work shifts. According to these results, unsupported kneeling was the most widely used knee posture in our sample (median 11.4 %, e.g. 55 min in a typical work shift of 480 min), followed by supported kneeling (15 min/480 min shift), sitting on heels (5 min), squatting (3 min), and crawling (0 min). The total mean exposure to the knee (=100 %) consisted mainly of unsupported kneeling (51.3 %), followed by supported kneeling (25.1 %), squatting (12.8 %), sitting on heels (9.5 %), and crawling (1.2 %).

### Exposure to the knee in different occupations and task modules

Based on the measured and extrapolated duration of knee-straining postures per work shift, the daily degree of exposure varied widely, as well as varying within an occupation. For example, daily time intervals of exposure to the knee within a single occupation could range from 0.3 to 60.9 % (screed layers) or from 0.0 to 88.9 % (parquet layers) (Table 3).

**Table 3:** Mean time proportions spent in the five knee-straining postures in 81 task modules of 16 occupations (*N* = 242 work shifts, *n* = examined work shift per task module)

Occupation	Task module	*n*	Total exposure (% work shift)	Squatting (% work shift)	Sitting on heels (% work shift)	Unsupported kneeling (% work shift)	Supported kneeling (% work shift)	Crawling (% work shift)
Floor layers	Installing carpets	6	48.2 (5.9)	0.3 (0.3)	4.7 (2.7)	23.1 (4.7)	16.6 (8.4)	3.5 (4.1)
	Carpet removal	3	44.5 (0.7)	0.8 (0.3)	5.1 (2.0)	18.6 (7.1)	17.1 (5.6)	2.9 (0.9)
	Preparation work	4	22.0 (23.0)	0.1 (0.1)	1.9 (2.7)	5.8 (4.6)	13.8 (16.1)	0.4 (0.5)
	Installing carpets (vehicles)	3	37.7 (15.2)	3.3 (4.3)	2.8 (2.4)	20.4 (5.5)	8.8 (4.6)	2.4 (4.0)
Installers	Preparing underfloor heating	3	65.8 (21.7)	2.8 (1.2)	8.9 (9.7)	32.6 (2.0)	20.7 (12.6)	0.9 (1.1)
	Installing underfloor heating	5	40.3 (14.8)	3.1 (5.5)	4.1 (3.0)	18.3 (6.6)	14.8 (16.1)	0.0 (0.1)
	Installing heating system	3	7.7 (4.7)	1.8 (1.4)	1.6 (2.8)	4.0 (3.5)	0.2 (0.4)	0.0 (0.0)
	Installing radiators	3	51.0 (5.2)	1.4 (1.8)	14.8 (16.3)	34.1 (10.6)	0.7 (0.2)	0.0 (0.0)
	Installing pipe	6	37.8 (12.6)	2.7 (2.8)	5.5 (6.2)	26.3 (14.1)	3.4 (4.0)	0.0 (0.0)
	Installing sewer pipe	2	52.3 (6.7)	7.9 (2.7)	7.0 (7.3)	32.9 (14.8)	4.6 (1.9)	0.0 (0.0)
	Installing concealed cistern	2	34.5 (26.0)	1.3 (0.4)	0.5 (0.7)	30.2 (21.4)	2.5 (3.5)	0.0 (0.0)
	Installing toilets and wash basins	4	41.5 (1.9)	2.5 (4.3)	5.8 (5.4)	28.1 (7.8)	5.2 (4.1)	0.0 (0.0)
	Installing roof flashing	4	20.3 (17.7)	11.1 (18.0)	0.1 (0.3)	6.3 (4.4)	2.8 (3.7)	0.0 (0.0)
	Installing gutters	3	5.7 (7.5)	0.2 (0.1)	0.0 (0.0)	2.6 (2.8)	2.8 (4.8)	0.0 (0.0)
	Installing PV-system (flat roof)	3	5.3 (5.0)	1.5 (1.2)	0.1 (0.2)	3.0 (3.3)	0.7 (1.2)	0.0 (0.0)
	Installing PV-system (steep roof)	2	25.6 (3.4)	2.0 (1.3)	1.4 (0.2)	15.6 (9.6)	6.7 (5.1)	0.0 (0.0)
Mould makers	Mould making	4	6.5 (3.0)	0.2 (0.3)	0.3 (0.2)	2.5 (0.8)	3.6 (3.0)	0.0 (0.1)
Painters and decorators	Preparing masonry painting	3	35.0 (21.4)	7.9 (6.0)	5.6 (5.6)	20.3 (13.6)	1.4 (1.7)	0.0 (0.0)
	Masonry painting	3	9.0 (5.2)	5.3 (6.9)	0.6 (1.1)	2.7 (1.4)	0.4 (0.6)	0.0 (0.0)
	Installing external wall insulation	5	8.9 (12.2)	4.5 (9.4)	2.3 (4.9)	2.1 (2.4)	0.1 (0.1)	0.0 (0.0)
	Wallpapering	3	24.2 (7.1)	1.6 (2.4)	6.3 (5.1)	15.5 (4.0)	0.7 (0.6)	0.0 (0.0)
	Interior painting (brush)	2	35.4 (8.5)	9.2 (11.7)	1.1 (0.6)	23.6 (5.8)	1.5 (2.1)	0.0 (0.0)
	Interior painting (paint roller)	1	3.0 (–)	1.7 (–)	0.0 (–)	1.3 (–)	0.0 (–)	0.0 (–)
	Painting a stairwell	2	14.0 (6.8)	1.5 (1.4)	5.1 (3.9)	7.3 (4.5)	0.1 (0.2)	0.0 (0.0)
Parquet layers	Laying strip parquet	3	74.1 (7.5)	0.6 (0.4)	2.2 (1.7)	58.5 (10.4)	12.7 (17.5)	0.2 (0.2)
	Laying mosaic parquet	8	52.4 (5.9)	2.6 (2.8)	3.0 (1.3)	28.6 (9.2)	18.1 (7.3)	0.1 (0.1)
	Sanding finish (grinding)	10	34.9 (14.2)	0.3 (0.4)	1.4 (1.4)	21.1 (13.2)	12.1 (7.9)	0.1 (0.1)
	Preparation work	2	2.5 (3.1)	0.3 (0.1)	0.0 (0.0)	2.3 (3.2)	0.0 (0.0)	0.0 (0.0)
	Installing board parquet (planks)	1	33.7 (–)	5.3 (–)	7.4 (–)	11.4 (–)	9.3 (–)	0.2 (–)
	Preparing strip parquet	3	0.0 (0.0)	0.0 (0.0)	0.0 (0.0)	0.0 (0.0)	0.0 (0.0)	0.0 (0.0)
	Installing base	1	61.8 (–)	0.5 (–)	5.4 (–)	29.2 (–)	26.1 (–)	0.5 (–)
Pavers	Laying interlocking paving stones	3	17.8 (3.1)	3.5 (5.4)	0.5 (0.9)	10.5 (6.2)	3.2 (3.1)	0.0 (0.0)
	Laying cobblestones	3	82.5 (5.9)	80.2 (2.5)	0.0 (0.0)	2.3 (4.0)	0.0 (0.0)	0.0 (0.0)
	Laying cobblestones (using stool)	1	0.0 (–)	0.0 (–)	0.0 (–)	0.0 (–)	0.0 (–)	0.0 (–)
Pipe layers	Sewer construction	3	2.0 (1.3)	0.8 (0.3)	0.1 (0.1)	0.8 (0.8)	0.3 (0.4)	0.0 (0.0)
	Pipe laying (welding)	3	13.9 (5.9)	2.3 (2.1)	0.8 (1.4)	7.2 (4.6)	3.5 (2.9)	0.1 (0.1)
	Pipe laying (PE welding)	2	21.9 (10.6)	0.1 (0.1)	4.3 (4.3)	16.1 (7.4)	1.4 (1.4)	0.0 (0.0)
	Digging	1	0.0 (–)	0.0 (–)	0.0 (–)	0.0 (–)	0.0 (–)	0.0 (–)
Ramp agents	Wide and narrow body aircrafts	3	5.8 (3.4)	0.4 (0.6)	1.9 (2.3)	1.8 (1.3)	1.6 (0.4)	0.1 (0.0)
	Narrow body aircrafts	5	17.4 (3.8)	0.1 (0.1)	2.6 (1.0)	9.1 (2.4)	5.0 (3.3)	0.6 (0.4)
Reinforcing ironworkers	Rebar tying	3	16.7 (12.6)	8.3 (3.1)	0.5 (0.9)	7.4 (11.9)	0.5 (0.9)	0.0 (0.0)
	Form working	3	14.2 (11.4)	5.1 (1.1)	0.5 (0.7)	5.6 (6.8)	3.0 (3.7)	0.0 (0.1)
Roofers (steep roofs)	Installing battens	4	4.2 (4.0)	0.3 (0.3)	0.1 (0.1)	2.9 (2.6)	0.9 (1.8)	0.0 (0.0)
	Installing insulation	2	48.9 (13.5)	2.6 (2.0)	1.0 (0.9)	36.8 (5.7)	8.2 (5.1)	0.2 (0.2)
	Installing roof tiles	3	7.2 (7.6)	0.5 (0.6)	1.3 (2.2)	3.5 (3.9)	1.9 (1.8)	0.1 (0.2)
	Installing plain tiles	4	27.2 (18.8)	2.0 (2.6)	0.7 (0.8)	17.4 (16.0)	7.2 (5.7)	0.0 (0.0)
	Slate roofing	2	48.7 (16.1)	0.3 (0.1)	3.1 (2.6)	29.2 (9.5)	16.1 (9.1)	0.0 (0.0)
	Mansard slate roofing	3	18.7 (8.3)	2.1 (2.5)	9.5 (5.2)	6.8 (5.9)	0.2 (0.2)	0.0 (0.0)
	Installing corrugated panels	3	7.0 (6.0)	2.7 (3.6)	0.3 (0.6)	3.8 (6.6)	0.2 (0.3)	0.0 (0.0)
	Reed roofing	3	3.7 (6.0)	0.1 (0.1)	0.0 (0.0)	3.6 (6.0)	0.0 (0.0)	0.0 (0.0)
	Reed removal	1	3.0 (–)	0.2 (–)	0.6 (–)	1.6 (–)	0.6 (–)	0.0 (–)
	Roof tile transport	1	2.8 (–)	0.3 (–)	0.0 (–)	1.6 (–)	0.9 (–)	0.0 (–)
	Wood framing work (carpenter)	1	14.6 (–)	0.3 (–)	0.2 (–)	7.1 (–)	6.9 (–)	0.1 (–)
Roofers (flat roofs)	Torch-on roofing	4	18.1 (10.9)	1.7 (3.0)	1.3 (1.5)	11.5 (6.5)	3.6 (2.4)	0.0 (0.1)
	Sealing roof to wall	2	64.7 (0.7)	0.4 (0.3)	3.5 (0.8)	39.9 (21.4)	20.8 (20.1)	0.0 (0.0)
	Installing PVC membranes	3	22.1 (17.4)	10.5 (14.5)	0.6 (0.6)	8.5 (4.7)	2.5 (3.7)	0.1 (0.1)
Screed layers (flowing screed)	Installing insulation	4	49.3 (7.3)	3.3 (3.8)	3.3 (2.9)	27.2 (12.4)	12.3 (8.4)	3.2 (2.6)
	Installing flowing screed	5	7.3 (6.5)	3.3 (4.7)	0.4 (0.9)	3.2 (3.2)	0.4 (0.7)	0.0 (0.0)
Screed layers (sand and cement screed)	Screeding the floor (team of 3)	3	52.2 (8.0)	0.4 (0.3)	2.1 (1.6)	14.0 (3.6)	35.4 (6.3)	0.2 (0.2)
	Screeding the floor (team of 2)	1	55.2 (–)	1.6 (–)	2.1 (–)	31.0 (–)	20.5 (–)	0.0 (–)
	Planing the screed (team of 3)	3	33.3 (13.6)	1.0 (0.9)	2.7 (1.9)	9.4 (6.7)	19.6 (11.8)	0.5 (0.4)
	Mixing the screed (team of 3)	2	0.4 (0.1)	0.0 (0.0)	0.0 (0.1)	0.3 (0.1)	0.0 (0.0)	0.0 (0.0)
	Mixing the screed (team of 2)	2	17.7 (2.5)	1.3 (0.3)	0.2 (0.1)	8.4 (0.1)	7.8 (2.1)	0.0 (0.0)
Shipyard workers	Welding	3	61.2 (33.9)	3.8 (4.0)	4.0 (5.6)	45.5 (28.4)	7.9 (8.0)	0.1 (0.1)
	Mechanic work	2	31.5 (10.7)	4.3 (4.0)	2.9 (0.3)	20.1 (1.0)	2.2 (2.7)	2.1 (2.8)
	Grinding	1	33.3 (–)	10.3 (–)	0.0 (–)	17.0 (–)	6.1 (–)	0.0 (–)
Stone layers	Staircase laying	5	29.7 (10.2)	11.0 (9.2)	3.3 (3.6)	14.6 (17.4)	0.9 (0.6)	0.0 (0.0)
	Cladding facades	5	16.2 (8.2)	7.3 (4.7)	0.1 (0.3)	8.1 (5.7)	0.6 (0.6)	0.0 (0.0)
	Setting floor tiles	3	32.8 (6.5)	1.8 (1.3)	1.4 (1.3)	15.7 (5.7)	13.9 (2.0)	0.0 (0.0)
	Vacuum lifter operator	1	1.4 (–)	0.9 (–)	0.0 (–)	0.1 (–)	0.5 (–)	0.0 (–)
	Stone layer with vacuum lifter	1	52.3 (–)	0.3 (–)	3.0 (–)	26.7 (–)	22.3 (–)	0.0 (–)
Tilers	Floor tiling (thin-bed method)	5	63.7 (9.3)	0.3 (0.3)	10.5 (2.5)	24.3 6.6	28.5 (5.6)	0.0 (0.1)
	Wall tiling (thin-bed method)	3	28.9 (16.7)	5.8 (5.3)	5.5 (3.4)	13.6 (9.0)	4.1 (2.0)	0.0 (0.0)
	Grouting floor tiles	2	66.7 (2.8)	7.3 (10.2)	11.9 (3.5)	17.3 (3.8)	29.7 (5.0)	0.5 (0.6)
	Grouting wall tiles	5	29.0 (5.7)	6.3 (7.3)	6.9 (6.3)	13.9 (7.6)	1.9 (1.8)	0.0 (0.0)
	Preparation work	2	27.3 (7.0)	0.3 (0.2)	2.9 (2.4)	19.1 (9.4)	4.9 (0.2)	0.2 (0.3)
	Floor tiling (thick bed method)	1	61.8 (–)	2.3 (–)	5.7 (–)	23.4 (–)	30.4 (–)	0.0 (–)
	Siliconing bath room	1	33.1 (–)	13.9 (–)	0.0 (–)	18.3 (–)	0.9 (–)	0.0 (–)
	Wall and floor tiling (thin bed)	1	48.3 (–)	0.0 (–)	7.8 (–)	32.6 (–)	7.8 (–)	0.0 (–)
Truck tarp makers	Producing truck tarps	5	21.9 (5.1)	3.6 (4.8)	0.4 (0.5)	13.1 (3.1)	2.0 (2.3)	2.9 (3.4)
Welders (container)	Welding partition walls	3	40.9 (12.1)	0.4 (0.4)	2.1 (2.4)	14.6 (17.5)	23.9 (8.7)	0.0 (0.0)

Values are mean values (standard deviations)

*PV* photovoltaic, *PE* polyethylene

There are some examples of task modules showing a relatively homogenous exposure to the knee per work shift, for example carpet removal [floor layers, total exposure 44.5 ± 0.7 % (*n* = 3 work shifts)], installing radiators [installers, 51.0 ± 5.2 % (*n* = 3)], or laying mosaic parquet [parquet layers, 52.4 ± 5.9 % (*n* = 8)]. In contrast, tasks with quite heterogeneous exposure to the knee were also measured, such as preparing masonry painting [painters, 35.0 ± 21.4 % (*n* = 3)] or the preparation work of floor layers [22.0 ± 23.0 % (*n* = 4)].

## Discussion

Our study covers a broad spectrum of occupations known for knee-straining activities and assessed the typical tasks. The results show that 75 % of occupational exposure to the knee was in the posture of kneeling and less than 25 % in sitting on heels, squatting, and crawling. This might be an important hint for the interpretation of self-reported exposure to the knee where subjects often fail to assess the duration they spent in different knee postures correctly (Ditchen et al. 2013). Despite this predominance of one posture, our findings illustrate huge variety of occupational exposure to the knee and the difficulty of quantifying this exposure by specific categories, for example job categories. Due to different work content, specific characteristics of construction sites and workplaces, and individual preferences of working postures, the spectrum of daily exposure within a single job can vary greatly: Parquet layers’ or installers’ percentage of time spent in knee-straining postures per day, for example ranged from 0.0 to 74.1 %, and 5.5 to 65.8 %, respectively (Table 3). Thus, our findings seem to be in line with the results of Tak et al. (2009) who stated that organisational features such as job categories cannot be regarded as homogenous exposure groups. The authors recommend that “exposures should be stratified by operation and task for the development of similar exposure groups”. Furthermore, our study focussed on task modules only involving kneeling and squatting. This is an important consideration for the reconstruction of average job-specific exposure profiles to the knee as there are usually other task modules without kneeling or squatting in all occupations. Documenting such activities for the examined occupations and describing the frequency of the examined task modules might be a potential way to develop a task exposure matrix (TEM). TEMs are described for various exposures, for example inspirable dusts and benzene soluble fractions by Benke et al. (2000). In contrast to this, in the field of ergonomic epidemiology, there have been some suggestions that assessment strategies focussing on occupations rather than tasks may be preferable (Mathiassen et al. 2005; Svendsen et al. 2005). But irrespective of the strategy selected, valid exposure data are still required. A parallel conducted comparison of our measuring data and workers’ self-reports (Ditchen et al. 2013) showed that subjects were not able to assess their time spent in knee-straining postures reliably, both immediately after the measurement and six months later. But on the other hand, workers were able to accurately remember the occurrence of different knee-straining postures while performing a specific task. Thus, there might be a chance of improving exposure assessment using measurement data in combination with interview data, a method, for example used in the research on Parkinson’s disease (Semple et al. 2004). As our pilot study showed, the adequate combination of selective measuring phases and diary information can be nearly as accurate as whole day measuring in the case of occupational knee-exposure.

With regard to the high variability of the exposure within a single task module, we found different reasons that may explain this. In many tasks, different working heights influenced workers’ posture, for example while working on scaffoldings, as do painters and roofers. A similar effect could be observed for roofers on steep roofs; the degree of the roof pitch strongly determined the workers’ postures (standing, “knee-supporting position” (Jensen et al. 2000b), or kneeling/squatting). Other factors that influenced the choice of posture included different structures on construction sites, different working techniques, and, last but not least, individual preferences.

It is difficult to compare our results with those of similar studies as only a few studies have been concerned with the daily exposure to the knee. In a Finnish study (Kivimäki et al. 1992) on knee disorders of carpet and floor layers and painters, 35 subjects performing different tasks were videotaped for a total time of 12 h. In this study, only short working sequences of between 33 and 102 min were analysed, without regard to breaks, preparation work, et cetera. By projecting these results onto a whole work shift, the comparison with our findings yielded agreements (e.g. parquet or floor layer, installing base: approx. 60 % of knee strain per day to approx. 62 % per day in our study) and strong disagreements (e.g. parquet or floor layer, installing mosaic parquet: approx. 90 % per day to approx. 52 % per day in our study). In accordance to our study, the authors found large task-specific differences in the degree of exposure within a job category; for example, floor layers’ percentage of kneeling and squatting ranged from 0 % (grinding) to approximately 90 % (installing mosaic parquet) of the observation time.

The importance of including all daily activities in the analysis of kneeling and squatting is made apparent in the studies of Jensen et al. in Denmark. In a first study, the authors videotaped floor layers and carpenters during short time sequences of three to 30 min (Jensen et al. 2000a, b). By extrapolating their findings on the duration of kneeling and squatting to a whole work shift, they stated an average daily percentage of time spent in these postures of approximately 56 % (floor layers) and 25 % (carpenters). In a second study, the authors videotaped each of four floor layers for an entire work shift and analysed the duration of kneeling, squatting, kneeling back on heels, and crawling tasks (Jensen et al. 2010). The average percentage of time spent in these postures was 41.0 % (SD = 7.5), which is consistent with our result of 39.0 % (SD = 16.3) from analysing all floor layers’ tasks measured in our study. As mentioned before, the analysis of only short working sequences may lead to overestimation of the real exposure.

The effects of mechanised equipment on physical load such as kneeling among screed layers (mentioned as floor layers) and pavers (mentioned as road workers) are mentioned in Dutch study by Burdorf et al. (2007). Knee-straining postures of 32 screed layers and 27 pavers were captured by an ambulant monitor using accelerometry. The authors found that screed layers working alone to produce a sand-cement floor were in kneeling and squatting postures for approximately 48 % of their work time, and screed layers working with the help of a hodman were in these postures for approximately 40 % of their work time. These results are consistent with our findings for screed layers screeding the floor (in a team of 3) with 52.2 % of knee-straining postures per day. In contrast, our results for pavers (or road workers) deviated from those of the Dutch study. While the researched German pavers laid the interlocking paving stones predominantly in a standing posture (approx. 18 % of knee-straining postures per day), the Dutch road workers preferred a kneeling position (approx. 48 % of knee-straining postures per day). In that, both the German and the Dutch road workers may have used different working techniques; these results illustrate again the problem of using job categories as homogenous exposure groups. Even if both groups had the same kind of working task, their exposure could only be assessed correctly by a detailed description of their actual working methods.

### Weaknesses and strengths

As we were performing a field-study at real construction sites, our study was subjected to some limitations, especially in the planning of measurements. As a result of various influences such as poor weather conditions or machine failures at the work sites, we were not able to measure each task module at least three times as planned (26 of 81 task modules (=32,1 %) were measured less than three times). This fact and the occasionally observed large between-subjects variability may limit the representativeness of our results.

We were only able to measure current working techniques. Different techniques of the past may have shown different exposure to the knee. This may be essential for epidemiological studies or in treatment of occupational diseases and must be considered in each individual case.

Nearly all measurements took place at large construction sites where the examined task modules were usually performed during an entire work shift. At smaller building lots, the extent of exposure may differ. As all study participants were male, we cannot give any statement on gender differences with respect to knee-straining postures.

All enterprises were approached and recruited by the German Statutory Accident Insurances, and all agreed to participate in the study. Thus, there might be a selection bias in recruiting the employees as they were chosen at running construction sites in the recruitment period. However, this effect might be reduced in that the 110 participating enterprises were spread all over Germany and recruited by more than 20 different persons.

Our study is characterised by an accurate and feasible method of posture capturing at real workplaces in various occupations. The detailed documentation of the examined work shifts permitted whole-shift analyses with respect to the daily exposure to the knee. As our validation analysis has shown, the combination of measuring data and information delivered by diaries or schedules can be a promising approach to obtain valid data with less resources being required. For this selective procedure, we consulted technical experts as detailed knowledge of the analysed tasks is essential.

## Conclusion

As knee-straining postures seem to vary to a great extent within a job category, we suggest assessing such activities task-specifically, both for preventive purposes and for exposure assessment. For the latter case, the use of task-based measurement data in combination with diary information may be a promising choice to find a compromise between valid information and cost efficiency.
